# Responsiveness and minimal important change of the Dutch Adult Strabismus-20 Questionnaire (Dutch-AS-20)

**DOI:** 10.1007/s11136-026-04348-z

**Published:** 2026-09-02

**Authors:** Fenna Burggraaf, Hinke Marijke Jellema, Martha J. Tjon-Fo-Sang, Ruth M. A. van Nispen, Ellen B. M. Elsman

**Affiliations:** 1 The Rotterdam Eye Hospital, Rotterdam, The Netherlands; 2 Amsterdam UMC location Vrije Universiteit Amsterdam, Ophthalmology, Boelelaan 1117, Amsterdam, The Netherlands; 3 Amsterdam Public Health, Quality of Care, Amsterdam, The Netherlands

**Keywords:** AS-20, Responsiveness, Minimal important change, Quality of life, Adult strabismus, Strabismus surgery

## Abstract

**Purpose:**

The Dutch Adult Strabismus-20 questionnaire (Dutch-AS-20) is a patient-reported outcome measure (PROM) developed to assess the strabismus-specific quality of life (QoL) in adult patients with strabismus in the Netherlands. This 18-item questionnaire comprises a psychosocial and visual function subscale. The aim of this prospective, multicenter, longitudinal study was to evaluate the responsiveness and determine the minimal important change (MIC) of the Dutch-AS-20 in patients undergoing strabismus surgery.

**Methods:**

Adult patients scheduled for strabismus surgery were enrolled. The Dutch-AS-20, EQ-5D-5L, Amblyopia and Strabismus Questionnaire (A&SQ), Diplopia Questionnaire (DQ) and two global rating of change (GRC) questions were administered pre- and/or postoperatively. We used a construct approach to assess the responsiveness, testing eight pre-specified hypotheses. We determined the MIC using adjusted anchor-based predictive modelling.

**Results:**

A total of 136 patients completed the questionnaires at baseline. All pre-specified hypotheses were met, indicating sufficient responsiveness of both subscales of the Dutch-AS-20. We estimated a MIC of 3.1 T-score points for the psychosocial subscale. We could not estimate the MIC for the visual function subscale due to a low correlation with the GRC question.

**Conclusion:**

Both subscales of the Dutch-AS-20 are sufficiently responsive to change in strabismus-specific QoL following strabismus surgery. These findings are an important step towards the use of the Dutch AS-20 in both research and clinical practice. Future research is needed to determine a more precise MIC and individual-level statistics for both subscales, in order to improve the interpretability of change scores for both clinical and research purposes.

## Introduction

Strabismus refers to any horizontal, vertical and/or torsional misalignment of the eyes. It can develop in both children and adults [1] and has considerable impact on patients’ quality of life (QoL) [2]. Adults with strabismus report lower self-confidence, self-esteem, self-image and higher levels of depression and anxiety compared to the general population [2–4]. The presence of strabismus can have a negative effect on social life and employment [4].

Strabismus can be treated surgically and non-surgically [4]. Strabismus surgery in adults is mainly performed to restore ocular alignment to enhance physical appearance and/or to treat diplopia (double vision) [2, 4]. To evaluate the results of strabismus surgery, patient-reported outcome measures (PROMs) are increasingly used in addition to clinical outcomes [5]. Incorporating PROMs into routine patient assessment and strabismus management allows for a more comprehensive understanding of treatment effectiveness and quality of care [6–8]. In addition, PROM data can facilitate shared decision-making and support patient-centered care [7, 9, 10].

The Adult Strabismus-20 questionnaire (AS-20) is a PROM developed by Hatt and colleagues in 2009 to assess the strabismus-specific QoL in adult patients with strabismus [11, 12]. The AS-20 is based on a reflective model that assumes that all items in a (sub)scale reflect a single underlying latent construct [13–15]. The first (English) version of the AS-20 consisted of 20 items scored on a 5-point frequency scale with the response options: never, rarely, sometimes, often and always [11]. Although other strabismus-specific questionnaires exist (e.g. The Amblyopia and Strabismus Questionnaire, A&SQ), the AS-20 is the most widely used and is considered the most suitable instrument for assessing QoL in adult patients with strabismus [5, 12, 16].

The AS-20 has been translated into Dutch in 2019. Item response theory (IRT) analysis showed that the Dutch-AS-20 benefitted from removal of 2 items. The 18-item Dutch-AS-20 consists of a psychosocial (item 1–10) and visual function (item 11–18) subscale. The reliability and validity of both subscales of the Dutch-AS-20 have recently been established using IRT analysis and through the evaluation of the internal consistency, test–retest reliability and construct validity. IRT analysis showed that measurement precision and targeting were appropriate for adult patients with low to moderate levels of psychosocial and visual functioning [12]. Both the original AS-20 and the Dutch-AS-20 are available on the Pediatric Eye Disease Investigative Group (Jaeb Center for Health Research) website (https://public.jaeb.org/pedig/view/Forms_AS20view/Forms_AS20).

However, before using the Dutch-AS-20 in clinical practice or for research purposes, it is important to gain insight into the responsiveness of the instrument and to determine the minimal important change (MIC) [17, 18]. Responsiveness refers to “the degree to which an instrument is able to measure change in the construct to be measured” [17]. The MIC represents the smallest within-person change in a construct that patients consider important [18–20]. The aim of this prospective, multicenter, longitudinal study was therefore to evaluate the responsiveness and determine the MIC of the Dutch-AS-20 in Dutch adult patients with strabismus undergoing strabismus surgery.

## Methods

The study was conducted according to the principles of the Declaration of Helsinki. The Medical Ethics Review Committee of Amsterdam University Medical Center (UMC) confirmed that under Dutch law, the Medical Research Involving Human Subjects Act did not apply to this study. Written informed consent was obtained from all participants prior to inclusion, ensuring compliance with Dutch legislation (Dutch Medical Treatment Contracts Act and General Data Protection Regulation), codes of conduct for medical research, and institutional regulations for the use of personal data. This study was reported according to the COnsensus-based standards for the Selection of health Measurement INstruments (COSMIN) reporting guideline for studies on measurement properties of PROMs version 2.0 [21].

### Study population

We consecutively recruited patients between September 2022 and January 2023 from the orthoptic departments of the Rotterdam Eye Hospital and Amsterdam UMC, the Netherlands. Patients with any type of strabismus were eligible for inclusion if they met the following criteria: (1) aged 18 years or older, (2) scheduled for strabismus surgery at the Rotterdam Eye Hospital or Amsterdam UMC, (3) able to read and understand Dutch, (4) best-corrected distance visual acuity ≥ 0.3 Snellen in the better eye, (5) no severe cognitive impairment, (6) absence of monocular diplopia and (7) no other ophthalmic surgery within 6 weeks prior to recruitment and throughout the duration of the study. We excluded patients with ocular myasthenia gravis due to the variability of the condition [22]. According to COSMIN criteria, a sample size of ≥ 100 subjects is considered very good for responsiveness studies [23]. For the determination of the MIC the sample size should be at least 100 subjects as well [18]. We therefore aimed to include a minimum of 125 patients (expected drop-out rate 20%).

### Clinical data

All patients underwent a full orthoptic evaluation at the first and/or second preoperative visit(s) and 2 months postoperatively. We retrieved the orthoptic diagnosis, pre- and postoperative direction and size of deviation, best-corrected distance visual acuity of both eyes, presence of amblyopia, history of strabismus surgery, presence of surgical complications and information about the use of prism glasses or occlusion of one eye from the patient files. Amblyopia was defined as a ≥ 2 line difference in best-corrected distance Snellen visual acuity between both eyes without an organic cause [24]. We used the alternating prism cover test or Maddox cross in combination with the alternating cover test to measure the angle of deviation in primary position with habitual correction (without prism) at near (30 cm) and distance fixation (2.5 m). Krimsky measurements were used in case of low visual acuity [25]. All measurements were performed at a maximum fixation distance of 2.5 m to ensure consistency across methods.

### Questionnaires

After each visit patients were asked to complete several questionnaires online through a web-based survey. A paper-and-pencil version of the questionnaires was available at patient’s request. Questionnaire data were used to assess the responsiveness and determine the MIC of the Dutch-AS-20. Besides the Dutch-AS-20 [12], the survey included questions regarding (socio)demographic characteristics, the Diplopia Questionnaire (DQ) [26, 27], the A&SQ [28, 29], the EQ-5D-5L [30] and two global rating of change (GRC) questions. Patients were asked to answer all items as if they were wearing their habitual refractive correction (without prisms or occlusion foil) [31].

####  The Dutch Adult Strabismus-20 questionnaire

The 18-item Dutch-AS-20 was used to assess the strabismus-specific QoL before and after strabismus surgery. For this study, we derived total subscale scores from the IRT model using the item parameters from the original validation study [12]. Subscale scores were expressed as T-scores, with a mean of 50 and a standard deviation of 10 in the original population. Higher scores indicate better psychosocial and visual functioning.

#### The Diplopia Questionnaire

The DQ was used to determine the level of diplopia and to assign patients to different subgroups pre- and postoperatively. The DQ measures the severity of diplopia in 7 gaze positions: when reading, looking straight ahead in the distance and when looking up, down, right, left or in any other gaze position. Patients were asked if they experienced diplopia in the past week and those who answered “yes” were asked to rate the frequency of diplopia in each gaze position (always, often, sometimes, rarely or never) [26, 27, 32]. The DQ demonstrated good test–retest reliability [26, 32]. For this study, we translated the DQ from English into Dutch using forward translation only given the straightforward and unambiguous nature of the DQ items.

#### The Amblyopia and Strabismus Questionnaire and the EQ-5D-5L

The A&SQ and the EQ-5D-5L were used as comparator instruments to assess the responsiveness of the Dutch-AS-20. The A&SQ consists of 26 items and was developed to measure the strabismus-specific QoL in adult patients with (childhood-onset) strabismus and/or amblyopia [28, 29]. The items are organized into 5 subscales: fear of losing the better eye (item 1–3), distance estimation (item 4–13), visual disorientation (item 14–16), double vision (item 17–21) and social contact and appearance (item 22–26). Item 1, 4 and 5 are scored on a two- or three-point frequency scale. The remaining items are scored on a five-point frequency scale [16]. All item scores were subsequently transformed to a 0–100 scale, with a higher score indicating better strabismus-specific QoL. The total score of the A&SQ was calculated as the mean of all item scores [33]. The A&SQ was originally developed and validated in Dutch and showed good discriminative validity [29]. The EQ-5D-5L is a generic health-related quality of life instrument for describing/valuing health [30]. It covers five dimensions of health: mobility, self-care, usual activities, pain/discomfort and anxiety/depression. There are five response categories (levels) for each question corresponding to: no problems, some problems, moderate problems, severe problems, and extreme problems/unable to. The EQ-5D-5L ends with a visual analogue scale which asks patients to rate their overall health that day (0–100 score) [30, 34]. We used the Dutch tariff for the EQ-5D-5L to calculate the health state of each patient ranging from 1 (full health) to 0 (death) [34]. The Dutch EQ-5D-5L has been validated across different patient groups [34, 35].

#### Global rating of change questions

Following the guidelines by Terwee and colleagues [18], we formulated two GRC questions about perceived change (one for each subscale of the Dutch-AS-20) to serve as an anchor to determine the MIC (see Appendix 1). An independent panel evaluated the face and content validity of the GRC questions to assess whether the GRC questions and the subscales of the Dutch-AS-20 relate to the same construct. The panel consisted of seven orthoptists and nine adult patients with strabismus attending the Rotterdam Eye Hospital. Face validity, relevance and comprehensiveness of the two GRC questions were evaluated by the panel using a 10-item survey (see Appendix 2). After minor adjustments, face and content validity of both GRC questions was considered sufficient. Two months postoperatively, patients rated their perceived change on both GRC questions.

### Statistical analysis

We used R and R studio version 4.2.1 and SPSS version 28.0.1.1 to perform all statistical analyses. Descriptive statistics were used to describe (socio)demographic and clinical characteristics of the study sample. We evaluated reasons for dropout and excluded patients from the analyses in case of missing responses on the questionnaires or missing orthoptic data.

### Responsiveness

We used a construct approach for testing the responsiveness of the Dutch-AS-20. We formulated eight hypotheses a priori, four for each subscale. Responsiveness was considered sufficient if at least 75% of the hypotheses were confirmed [19, 36]. Two types of hypotheses were tested: (1) comparison with other outcome measurement instruments and (2) comparison between subgroups [36].

#### Comparison with other outcome measurement instruments

The first hypothesis for each subscale concerned the expected magnitude and direction of correlations between change scores on the Dutch-AS-20 subscales and change scores on the A&SQ and the EQ-5D-5L. Change scores on subscales of the Dutch-AS-20 and A&SQ (measuring a similar construct) were expected to correlate higher than change scores on subscales of the Dutch-AS-20 and EQ-5D-5L (measuring a related but dissimilar construct). Following the advice of Mokkink and colleagues, we chose to formulate hypotheses about the expected relative rather than absolute magnitude of change, given that there is no clear expectation about how much change is expected [36]. Spearman’s correlations were computed to determine the strength and direction of the association.

#### Comparison between subgroups

The other six hypotheses concerned the expected differences in change scores on subscales of the Dutch-AS-20 between: (1) patients receiving no intervention and patients undergoing strabismus surgery (i.e. improved patients according to clinical criteria), (2) patients receiving no intervention and sufficiently improved patients after strabismus surgery according to the GRC questions and (3) between successfully aligned patients (i.e. responders) with and without preoperative diplopia (examined by the DQ) [31, 36]. The mean change score was divided by the standard deviation (SD) of this change score to calculate the effect size (ES) otherwise known as the standardized response mean (SRM) [19]. More detailed information on the definitions of the different subgroups can be found in Appendix 3.

### Minimal important change

We determined the MIC in relation to the smallest detectable change (SDC) to interpret change scores on both subscales of the Dutch-AS-20. To assess if both GRC questions were suitable for estimating MIC values, the correlation between the change score of the subscales of the Dutch-AS-20 and the corresponding GRC question was determined. The correlation should be at least 0.30 to establish the validity of the GRC questions [18].

We used anchor-based MIC predictive modelling (MIC_predict_) to estimate the MIC using logistic regression analysis. The change score on each subscale was used as the independent variable and the group variable (improved vs not improved on the GRC questions) as the dependent variable [18, 20]. Patients who reported at least “a little better” or more were classified as improved, whereas the remainder of the patients were classified as not improved. Subsequently, the 95% confidence interval around the MIC was calculated [18]. We used the adjusted MIC predictive modelling method to correct for bias when the percentage of improved patients was different from 50% [18].

The SDC was calculated using the following formula: ${SDC}_{consistency} = 1.96 \times \surd 2 \times {SEM}_{consistency}$. The standard error of measurement (SEM), used to determine the SDC, was based on test–retest parameters (intraclass correlation coefficients, ICC_3,1_) calculated in the previous study (${SEM}_{consistency}= (SD) \times (\sqrt{1}-ICC))$, SD refers to the SD_pooled_ of the study sample) [12, 37]. Ideally, the MIC should exceed the SDC [19].

## Results

### Patient characteristics

A total of 179 patients were invited to participate in the study, of which 176 patients gave their informed consent. Out of these 176 patients, 136 (77%) filled out and returned the questionnaires at baseline (preoperatively). Twenty-two patients no longer wanted to participate, in 11 patients strabismus surgery was cancelled and 7 patients were lost to follow-up. There were no missing responses on the questionnaires or missing orthoptic data. Table 1 shows the clinical and (socio)demographic characteristics of the total sample at baseline. One hundred and thirteen patients (83% of baseline respondents) had undergone strabismus surgery during the course of the study. They completed the questionnaires again after a median postoperative period of 2 months (range 43–162 days). Median pre- and postoperative (subscale) scores of the Dutch-AS-20, A&SQ and the EQ-5D-5L can be found in Table 2. According to the clinical outcome criteria, 42 (37%) patients were classified as responder, 26 (23%) as partial responder and 45 (40%) as non-responder two months postoperatively. Eight (7%) patients experienced minor surgical complications (corneal astigmatism (n = 2), conjunctival inclusion cyst (n = 1), abduction limitation (n = 1), keratoconjunctivitis sicca (n = 1) and conjunctival pyogenic granulomas (n = 3). 

Responsiveness

The results in relation to the hypotheses are presented in Table 3. All results were in accordance with the predefined hypotheses, indicating sufficient responsiveness of both subscales of the Dutch-AS-20 two months following strabismus surgery. 

**Table 1:** Patient characteristics at baseline (preoperative) (N = 136)

Age in years, median (range)	53 (18–79)
Sex assigned at birth, N (%)	
Male	56 (41)
Female	80 (59)
Place of residence, N (%)	
Municipality of Amsterdam, Rotterdam or The Hague	47 (35)
North Holland, Utrecht or South Holland (excl. above municipalities)	52 (38)
Groningen, Friesland or Drenthe	2 (1)
Overijssel, Gelderland or Flevoland	12 (9)
Zeeland, North Brabant or Limburg	23 (17)
Education level, N (%)	
Primary	4 (3)
Secondary	75 (55)
Higher	53 (39)
Other	4 (3)
Currently having a paid (part-time) job, N (%)	91 (67)
Currently doing unpaid/voluntary work, N (%)	11 (8)
Financial situation, N (%)	
Usually enough money	97 (71)
Just enough money	34 (25)
Not enough money	4 (3)
No answer	1 (1)
Aetiology of strabismus, N (%)	
Childhood onset or idiopathic	73 (54)
Neurogenic	25 (18)
Mechanical	31 (23)
Sensory	7 (5)
Direction of deviation*, N (%)	
Esodeviation	52 (38)
Exodeviation	55 (40)
Vertical deviation	29 (22)
Diplopia, N (%)	90 (66)
Snellen distance visual acuity with habitual correction best eye, median (range)	1.0 (0.60–1.60)
Snellen distance visual acuity with habitual correction worse eye, median (range)	0.91 (LP-1.60)
Presence of amblyopia, N (%)	23 (17)
Use of prism glasses, N (%)	25 (18)
Use of occlusion, N (%)	5 (4)
History of strabismus surgery, N (%)	70 (52)
Comorbidity, N (%)	58 (43)
Ocular comorbidity, N (%)	24 (18)

Dutch-AS-20: 18-item Dutch Adult Strabismus-20 questionnaire, N: number of patients, LP: light perception,

^*^ Classification by the largest deviation in case of multi-planar deviations

**Table 2:** Pre- and postoperative scores for the Dutch-AS-20, A&SQ and the EQ-5D-5L

Instrument	Preoperative	Postoperative
*Dutch-AS-20 score, median T-score points (IQR)*		
Psychosocial subscale	45.1 (38.8–50.8)	45.6 (40.7–52.0)
Visual function subscale	49.2 (42.7–55.4)	53.3 (48.1–58.3)
*A&SQ score, median sum score (IQR)*		
Social contact and appearance subscale	56.3 (37.5–87.5)	75 (50–100)
Fear of losing the better eye, distance estimation, visual disorientation and double vision subscales	63.3 (51.5–81.3)	75 (62.5–83.3)
*EQ-5D-5L, median Index Score (IQR)*	0.90 (0.82–1.00)	0.93 (0.86–1.00)

A&SQ: Amblyopia and Strabismus Questionnaire, Dutch-AS-20: 18-item Dutch Adult Strabismus-20 questionnaire, IQR; interquartile range

**Table 3:** Hypotheses for the responsiveness of the Dutch-AS-20

Hypotheses		Results	Confirmed
Psychosocial subscale Dutch-AS-20			
1	The correlation between change scores on the psychosocial subscale of the Dutch-AS-20 and the social contact and appearance subscale of the A&SQ is at least 0.1 higher than the correlation between change scores on the psychosocial subscale of the Dutch-AS-20 and the EQ-5D-5L (N = 113)	Correlations r_s_ = 0.48 vs r_s_ = 0.29 Difference: 0.19	Yes
2	The mean difference in change scores for the psychosocial subscale of the Dutch-AS-20 is greater for patients after strabismus surgery (clinically improved, N = 68) compared to patients receiving no intervention (N = 73; difference between groups ≥ 3 T-score)	Change scores T = 1.79 vs T = −1.33 Mean difference: T = 3.12	Yes
3	The mean difference in change scores for the psychosocial subscale of the Dutch-AS-20 is greater for importantly improved patients after strabismus surgery (according to the GRC question; N = 68) compared to patients receiving no intervention (N = 73; difference between groups ≥ 3 T-score)	Change scores T = 2.88 vs T = −1.33 Mean difference: T = 4.21	Yes
4	In successfully aligned patients (responders) without preoperative diplopia (N = 13) the SRM is higher on the psychosocial subscale of the Dutch-AS-20 than on the visual function subscale (difference in SRM ≥ 0.2)	SRM 0.40 vs −0.01 Difference: 0.41	Yes
Visual function subscale Dutch-AS-20			
5	The correlation between change scores on the visual function subscale of the Dutch-AS-20 and the fear of losing the better eye, distance estimation, visual disorientation and double vision subscales of the A&SQ is at least 0.1 higher than the correlation between change scores on the visual function subscale of the Dutch AS-20 and the EQ-5D-5L (N = 113)	Correlations r_s_ = 0.63 vs r_s_ = 0.25 Difference: 0.38	Yes
6	The mean difference in change scores for the visual function subscale of the Dutch-AS-20 is greater for patients after strabismus surgery (clinically improved, N = 68) compared to patients receiving no intervention (N = 73; difference between groups ≥ 3 T-score)	Change scores T = 4.49 vs T = −1.16 Mean difference: T = 5.65	Yes
7	The mean difference in change scores of the visual function subscale of the Dutch-AS-20 is greater for importantly improved patients after strabismus surgery (according to the GRC question; N = 63) compared to patients receiving no intervention (N = 73; difference between groups ≥ 3 T-score)	Change scores T = 5.07 vs T = −1.16 Mean difference: T = 6.23	Yes
8	In successfully aligned patients (responders) with preoperative diplopia (N = 29) the SRM is higher on the visual function subscale of the Dutch-AS-20 than on the psychosocial subscale (difference in SRM ≥ 0.2)	SRM 0.79 vs 0.24 Difference: 0.55	Yes
	Total amount of hypotheses confirmed		**8/8**

A&SQ: Amblyopia and Strabismus Questionnaire, Dutch-AS-20: 18-item Dutch Adult Strabismus-20 questionnaire, GRC: global rating of change, SRM; standardized response mean

### Minimal important change

The correlation between the change score on the visual function subscale of the Dutch-AS-20 and the corresponding GRC question was r_s_ = 0.14, which is below the threshold of ≥ 0.3. As such, the data were not suitable for estimating the MIC of the visual function subscale. The correlation between the change score on the psychosocial subscale of the Dutch-AS-20 and the corresponding GRC question was r_s_ = 0.39, confirming the validity of the anchor.

A total of 74 (66%) patients showed improvement on the psychosocial subscale of the Dutch-AS-20 two months postoperatively. Data from these patients were used to determine the MIC for improvement of the Dutch-AS-20 psychosocial subscale. Of these 74 patients, 50 (68%) rated themselves as improved and 24 (32%) as not improved based on the GRC question. Because of the uneven distribution, we used the adjusted MIC predictive modelling method to correct for bias. The adjusted MIC_predict_ for the psychosocial subscale of the Dutch-AS-20 was 3.1 T-score points (95% CI: 2.0–6.1). The SDC of the psychosocial subscale of the Dutch-AS-20 was smaller (2.7 T-score points) than the MIC_predict_, allowing clinically important changes to be distinguished from measurement error [19, 38].

## Discussion

In this study we evaluated the responsiveness and determined the MIC of the Dutch-AS-20, a PROM measuring strabismus-specific QoL in adult patients with strabismus. We found that all predefined hypotheses were met, indicating sufficient responsiveness of both subscales of the Dutch-AS-20 following strabismus surgery. Using adjusted anchor-based predictive modelling, we estimated a MIC of 3.1 T-score points for the psychosocial subscale of the Dutch-AS-20. We could not estimate the MIC for the visual function subscale due to a low correlation with the GRC question.

The AS-20 is available in a variety of languages [11, 39–48], yet research on the responsiveness and MIC is limited. There is only one study reporting on the responsiveness of the original English AS-20. This study tested the hypothesis that the AS-20 (subscale) score would change more in patients who were classified as successfully aligned compared to those who were unsuccessfully aligned after strabismus surgery. Eleven (61%) of the comparisons were in line with the hypothesis, which the authors considered to be enough evidence to support the responsiveness of the AS-20 [31]. Direct comparison with our findings is difficult because the hypotheses tested in this study differed, but both studies provide evidence that the AS-20 is responsive to changes after strabismus surgery. However, it is important to keep in mind that the assessment of responsiveness is an ongoing process and cannot be considered ‘proven’ [19, 36]. There have been no previous studies that estimated the MIC of the AS-20, underscoring the novelty of our results.

The MIC found in this study can be used for different purposes. In research, it can serve as a threshold to determine the number of improved patients following strabismus surgery. In clinical practice, it can be used to determine the number of improved patients following strabismus surgery in order to inform future patients about the expected effect of surgery on psychosocial functioning. However, it is important to note that the estimated MIC value is derived from a group of patients and may not apply to each individual patient. It is therefore advisable to use the MIC as a probabilistic value in individual patients (i.e. if a patient has changed ≥ 3.1 T-score points on the psychosocial subscale of the Dutch-AS-20 after strabismus surgery, it is more likely that he or she has importantly improved vs not importantly improved). Information on the MIC can be used as a starting point for a conversation about the effect of treatment and to support shared decision making [18].

This study has several strengths. Consistent with the COSMIN guidelines, we tested prespecified hypotheses to assess responsiveness, including both the expected direction and magnitude of correlations and differences in change scores [23, 36]. This rigorous approach enhances the robustness and credibility of the findings. Furthermore, we determined the MIC using an anchor-based method rather than the commonly used distribution-based methods. The anchor-based approach is more appropriate because it links changes in the instrument of interest to an anchor question reflecting *important* change, whereas distribution-based methods capture only minimal *detectable* change. Additionally, we applied the MIC predictive modelling method instead of the Receiver Operating Characteristic (ROC) curve method, as it provides greater precision and allows correction for bias when the proportion of improved patients deviates from 50% [18]. Finally, the high response rate and lack of missing data enhance the representativeness and validity of our results.

However, limitations should also be acknowledged. First, the subgroup used to estimate the MIC (N = 74) was smaller than the recommended 100 patients, and the proportion of improved patients deviated from 50%. These factors may have reduced the precision of the adjusted MIC, resulting in a wide confidence interval. Second, our results are limited to the MIC for improvement; the MIC for deterioration could not be determined due to an even smaller sample size (N = 39), preventing reliable estimation. Third, more than 15% of the patients scored at the upper end of the psychosocial subscale preoperatively (T-score ≥ 50), causing a ceiling effect that may have pushed the estimated MIC downward [20]. Fourth, the MIC of the visual function subscale could not be determined due to the low correlation between changes in the visual function subscale and the corresponding GRC question. The GRC question assesses perceived changes in ‘daily functioning’, whereas the visual function subscale of the Dutch-AS-20 specifically address ‘visual functioning’. In retrospect, we can conclude that the GRC question and the visual function subscale of the Dutch-AS-20 do not measure the same latent construct. This highlights a known limitation of anchor-based MIC methods – namely, that their results depend on the validity of the single anchor question [18, 19]. Finally, it is important to acknowledge that both responsiveness and the MIC are dependent on the setting and patient population in which the instrument is being used. This study was conducted in specialized centers providing diagnostics and treatment to complex strabismus patients referred from hospitals across the Netherlands. Consequently, this patient group may differ from those treated in other Dutch hospitals, potentially limiting the generalizability of our findings [49].

Given these points, the present study offers only limited insight into the interpretability of change scores on both subscales of the Dutch-AS-20. As the methodology for determining the MIC continues to evolve [50–52], future studies should apply either new methods or anchor-based methods using multiple anchors [53] in sufficiently large and representative patient samples to establish more precise MIC values for both improvement and deterioration for each Dutch-AS-20 subscale. Furthermore, the estimation and use of individual-level statistics may be a valuable addition to evaluate the significance of change at the individual level [54]. Until such evidence is available, care must be taken when interpreting change scores in both clinical and research settings. The findings regarding responsiveness contribute to the growing body of evidence on the psychometric properties of the Dutch-AS-20 and provide a foundation for its implementation in clinical practice. Future research focusing on the optimal strategies for implementing the Dutch-AS-20 in clinical practice is recommended.

## Conclusion

In conclusion, this study showed that both subscales of the Dutch-AS-20 are sufficiently responsive to change in strabismus-specific QoL in adult patients undergoing strabismus surgery. These findings are an important step towards the use of the Dutch AS-20 in both research and clinical practice. The MIC of the psychosocial subscale was estimated to be 3.1 T-score points (95% CI 2.0–6.1), whereas the MIC for the functional subscale could not be determined. Future research is needed to determine a more precise MIC and individual-level statistics for both subscales to improve the interpretability of change scores for both clinical and research purposes.

## Data Availability

The dataset generated and analyzed during the current study are not publicly available due to privacy and ethical restrictions. However, anonymized data may be obtained from the corresponding author upon reasonable request. All requests will be evaluated to ensure compliance with ethical approvals and the intended use of the data.

## Appendix 1. Global rating questions for the psychosocial (1) and visual function (2) subscale of the Dutch-AS-20

**Table:**

*Eye misalignment or double vision can affect self-confidence and daily functioning. We would like to know to what extent this has changed. Please answer the questions based on your experiences during the last week*	
(1) To what extent has your self-confidence changed compared to before squint surgery?	Ο Very much deteriorated Ο Much deteriorated Ο A little deteriorated Ο No change Ο A little improved Ο Much improved Ο Very much improved
(2) To what extent has your daily functioning changed compared to before squint surgery?	Ο Very much deteriorated Ο Much deteriorated Ο A little deteriorated Ο No change Ο A little improved Ο Much improved Ο Very much improved

Please note that this is not an official translation, as the questions were originally administered in Dutch.

## Appendix 2. Survey for panel to asses face and content validity of the global rating of change (GRC) questions

**Table:**

*Survey orthoptists*		
Introduction	We would like to determine the minimal important change (MIC) for both subscales of the Dutch-AS-20. This is the smallest change in the Dutch AS-20 score that patients perceive as important. To determine this, we plan to use the anchor-based method. This method uses an anchor question to assess the degree of change experienced by the patient. For the Dutch AS-20, two anchor questions have been formulated: one for the psychosocial subscale and one for the visual functional subscale. I would like to ask you to assess these anchor questions and judge the comprehensiveness, relevance and face validity Both subscales of the Dutch-AS-20 and both GRC questions were shown at the beginning of the survey. *Items 1 to 5 were administered separately for each of the two GRC questions	
1.	In your opinion, do the anchor question cover what the original questions of the *psychosocial/visual function** subscale of the Dutch AS-20 are assessing?	Yes / No If not, could you please explain why?
2.	Do you believe patients may find it difficult to answer the anchor questions?	Yes / No If yes, could you please explain why?
3.	Do you find the anchor questions unclear or confusing?	Yes / No If yes, could you please explain why?
4.	Are there any words used that might be difficult to understand?	Yes / No If yes, could you please explain why?
5.	Would you have formulated the anchor questions differently?	Yes / No If yes, could you please explain why?
*Survey patients*		
Introduction	The presence of strabismus (misalignment of the eyes) can affect your daily life (quality of life). A questionnaire can be used to measure how much strabismus impacts your quality of life. To make sure this questionnaire is good and measures what it’s supposed to, we need to check if it can detect relevant improvements or deteriorations in patients. To do this, different anchor questions are used. We want to be sure these questions are asked in the right way, and for that, we’d like your help. Would you be willing to fill out the questionnaire? Both GRC questions were shown at the beginning of the survey. Items 1 to 5 were administered separately for each of the two GRC questions	
1.	Did you have difficulty answering this question?	Yes, No If yes, could you please explain why?
2.	Did you find the question unclear or confusing?	Yes, No If yes, could you please explain why?
3.	Were there any words used that you found difficult to understand?	Yes / No If yes, which words?
4.	Did you find the way this question was phrased in any way disturbing or offensive?	Yes No If yes, could you please explain why?
5.	How would you ask the question?	

Please note that this is not an official translation, as the questions were originally administered in Dutch.

## Appendix 3. Definition different subgroups

**Table:**

Responder, partial responder or non-responder	Patients will be classified into three categories based on surgical outcomes 2 months following strabismus surgery (clinical criteria): responders, partial responders or non-responders. For responders all of the following criteria have to be met: (1) angle of strabismus at near and distance fixation ≤ 10Δ in primary position, (2) diplopia in primary position and reading was present never or rarely (examined by the DQ [26, 27]) and (3) no requirement for prism, occlusion or Bangerter foil. For partial-responder all of the following criteria have to be met: (1) the angle of strabismus at near and distance fixation was ≤ 15Δ in primary position, (2) diplopia in primary position and reading was present never, rarely or sometimes (examined by the DQ [26, 27]) and (3) no requirement for prism, occlusion or Bangerter foil. Patients were classified as non-responder if any one of the following criteria were met: (1) angle of strabismus at near and/or distance fixation > 15Δ in primary position, (2) diplopia in primary position and reading was present often or always (examined by the DQ [26, 27]) and (3) using prism, occlusion or Bangerter foil
Improved patients according to clinical criteria	Responders and partial-responders will be classified as improved patients according to clinical criteria [3, 26, 31]
Importantly improved group according to the GRC question	Patients answering the GRC question with “a little better” or more will be included in the importantly improved group [18]
Patients receiving no intervention	Patients receiving no intervention were patients on the waiting list (i.e. change score subscales Dutch-AS-20 first and second preoperative orthoptic assessment)

DQ: The Diplopia Questionnaire, GRC: global rating of change.

## References

[1] Martinez-Thompson, J. M., et al. (2014). Incidence, types and lifetime risk of adult-onset strabismus. *Ophthalmology,**121*(4), 877–882. 10.1016/j.ophtha.2013.10.030 PMC4321874 24321142

[2] McBain, H. B., et al. (2014). The impact of strabismus on quality of life in adults with and without diplopia: A systematic review. *Survey of Ophthalmology,**59*(2), 185–191. 10.1016/j.survophthal.2013.04.001 24359806

[3] McBain, H. B., et al. (2016). Does strabismus surgery improve quality and mood, and what factors influence this? *Eye,**30*(5), 656–667. 10.1038/eye.2016.70 PMC4869149 27126298

[4] MacKenzie, K., et al. (2016) Psychosocial interventions for improving quality of life outcomes in adults undergoing strabismus surgery. Cochrane Database of Systematic Reviews, 5. 10.1002/14651858.CD010092.pub4 PMC9250695 27171652

[5] Arblaster, G., et al. (2024). Strabismus surgery for psychosocial reasons: A literature review. *British & Irish Orthoptic Journal,**20*(1), 107–132. 10.22599/bioj.352 PMC11049605 38681188

[6] Hatt, S. R., et al. (2007). The effects of strabismus on quality of life in adults. *American Journal of Ophthalmology,**144*(5), 643–647. 10.1016/j.ajo.2007.06.032 PMC2241762 17707329

[7] Desomer, A., et al. *Use of patient-reported outcome and experience measures in patient care and policy*. 2018 KCE Reports 303. D/2018/10.273/40.: Health services research (HSR) Brussels: Belgian Health Care Knowledge Centre (KCE)

[8] Gunton, K. B. (2014). Impact of strabismus surgery on health-related quality of life in adults. *Current Opinion in Ophthalmology,**25*(5), 406–410. 10.1097/ICU.0000000000000087 25029092

[9] Ye, G., et al. (2020). A decision aid to facilitate informed choices among cataract patients: A randomized controlled trial. *Patient Education and Counseling*. 10.1016/j.pec.2020.10.036 33191060

[10] Ministerie van Volksgezondheid, W.e.S. *Ontwikkeling uitkomstgerichte zorg 2018–2022*. 2018 [cited 2021; Available from: https://www.rijksoverheid.nl/documenten/rapporten/2018/07/02/ontwikkeling-uitkomstgerichte-zorg-2018-2022.

[11] Hatt, S. R., et al. (2009). Development of a quality-of-life questionnaire for adults with strabismus. *Ophthalmology,**116*(1), 139–144. 10.1016/j.ophtha.2008.08.043 PMC2817952 19019449

[12] Burggraaf, F., et al. (2021). Psychometric properties of the Dutch version adult strabismus-20 questionnaire (AS-20). *Ophthalmic and Physiological Optics*. 10.1111/opo.12865 34392553

[13] Prinsen, C. A. C., et al. (2018). COSMIN guideline for systematic reviews of patient-reported outcome measures. *Quality of Life Research,**27*(5), 1147–1157. 10.1007/s11136-018-1798-3 PMC5891568 29435801

[14] Terwee, C. B., et al. (2018). COSMIN methodology for evaluating the content validity of patient-reported outcome measures: A Delphi study. *Quality of Life Research,**27*(5), 1159–1170. 10.1007/s11136-018-1829-0 PMC5891557 29550964

[15] Mokkink, L. B., et al. (2018). COSMIN risk of bias checklist for systematic reviews of patient-reported outcome measures. *Quality of Life Research,**27*(5), 1171–1179. 10.1007/s11136-017-1765-4 PMC5891552 29260445

[16] van de Graaf, E. S., et al. (2017). Differences in quality-of-life dimensions of adult strabismus quality of life and amblyopia & strabismus questionnaires. *Graefe’s Archive for Clinical and Experimental Ophthalmology,**255*(9), 1851–1858. 10.1007/s00417-017-3694-x PMC5554281 28555418

[17] Terwee, C. B., et al. (2007). Quality criteria were proposed for measurement properties of health status questionnaires. *Journal of Clinical Epidemiology,**60*(1), 34–42. 10.1016/j.jclinepi.2006.03.012 17161752

[18] Terwee, C. B., et al. (2021). Minimal important change (MIC): A conceptual clarification and systematic review of MIC estimates of PROMIS measures. *Quality of Life Research,**30*(10), 2729–2754. 10.1007/s11136-021-02925-y PMC8481206 34247326

[19] de Vet, H. C. W., et al. (2011). *Measurement in medicine: a practical guide. Practical guides to biostatistics and epidemiology*. Cambridge University Press.

[20] Terluin, B., et al. (2015). Minimal important change (MIC) based on a predictive modeling approach was more precise than MIC based on ROC analysis. *Journal of Clinical Epidemiology,**68*(12), 1388–1396. 10.1016/j.jclinepi.2015.03.015 25913670

[21] Gagnier, J. J., et al. (2025). COSMIN reporting guideline for studies on measurement properties of patient-reported outcome measures: Version 2.0. *Quality of Life Research,**34*(7), 1901–1911. 10.1007/s11136-025-03950-x 40153128

[22] Leske, D. A., Hatt, S. R., & Holmes, J. M. (2010). Test-retest reliability of health-related quality-of-life questionnaires in adults with strabismus. *American Journal of Ophthalmology,**149*(4), 672–676. 10.1016/j.ajo.2009.11.004 PMC2859184 20138603

[23] Mokkink, L.B. et al. (2019). *COSMIN study design checklist for patient-reported outcome measurement instruments*.

[24] Li, T., Qureshi, R., & Taylor, K. (2019). Conventional occlusion versus pharmacologic penalization for amblyopia. *Cochrane Database of Systematic Reviews,**8*, Article Cd006460. 10.1002/14651858.CD006460.pub3 PMC6713317 31461545

[25] Hatt, S. R., et al. (2018). Factors associated with failure of adult strabismus-20 questionnaire scores to improve following strabismus surgery. *JAMA Ophthalmology,**136*(1), 46–52. 10.1001/jamaophthalmol.2017.5088 PMC5833608 29167898

[26] Holmes, J. M., et al. (2013). Quantifying diplopia with a questionnaire. *Ophthalmology,**120*(7), 1492–1496. 10.1016/j.ophtha.2012.12.032 PMC3895465 23531348

[27] Holmes, J. M., Leske, D. A., & Kupersmith, M. J. (2005). New methods for quantifying diplopia. *Ophthalmology,**112*(11), 2035–2039. 10.1016/j.ophtha.2005.06.013 16185766

[28] van de Graaf, E. S., et al. (2007). Amblyopia and strabismus questionnaire (A&SQ): Clinical validation in a historic cohort. *Graefe’s Archive for Clinical and Experimental Ophthalmology,**245*(11), 1589–1595. 10.1007/s00417-007-0594-5 17549509

[29] van de Graaf, E. S., et al. (2004). Amblyopia & strabismus questionnaire: Design and initial validation. *Strabismus,**12*(3), 181–193. 10.1080/09273970490491196 15370526

[30] Herdman, M., et al. (2011). Development and preliminary testing of the new five-level version of EQ-5D (EQ-5D-5L). *Quality of Life Research,**20*(10), 1727–1736. 10.1007/s11136-011-9903-x PMC3220807 21479777

[31] Hatt, S. R., Leske, D. A., & Holmes, J. M. (2010). Responsiveness of health-related quality-of-life questionnaires in adults undergoing strabismus surgery. *Ophthalmology,**117*(12), 2322–2328. 10.1016/j.ophtha.2010.03.042 PMC2997934 20832120

[32] Mansukhani, S. A., et al. (2019). Test-retest reliability of the revised diplopia questionnaire. *Journal of AAPOS,**23*(6), 319.e1-319.e5. 10.1016/j.jaapos.2019.08.277 PMC6925322 31655115

[33] Felius, J., et al. (2007). The amblyopia and strabismus questionnaire: English translation, validation, and subscales. *American Journal of Ophthalmology,**143*(2), 305–310. 10.1016/j.ajo.2006.09.046 17157801

[34] Versteegh, M. M., et al. (2016). Dutch tariff for the five-level version of EQ-5D. *Value in Health,**19*(4), 343–352. 10.1016/j.jval.2016.01.003 27325326

[35] Janssen, M. F., et al. (2013). Measurement properties of the EQ-5D-5L compared to the EQ-5D-3L across eight patient groups: A multi-country study. *Quality of Life Research,**22*(7), 1717–1727. 10.1007/s11136-012-0322-4 PMC3764313 23184421

[36] Mokkink, L., Terwee, C., & de Vet, H. (2021). Key concepts in clinical epidemiology: Responsiveness, the longitudinal aspect of validity. *Journal of Clinical Epidemiology*. 10.1016/j.jclinepi.2021.06.002 34116141

[37] Mokkink, L. B., et al. (2020). COSMIN risk of bias tool to assess the quality of studies on reliability or measurement error of outcome measurement instruments: A Delphi study. *BMC Medical Research Methodology,**20*(1), 293. 10.1186/s12874-020-01179-5 PMC7712525 33267819

[38] Terwee, C. B., et al. (2009). Linking measurement error to minimal important change of patient-reported outcomes. *Journal of Clinical Epidemiology,**62*(10), 1062–1067. 10.1016/j.jclinepi.2008.10.011 19230609

[39] Fossum, P., et al. (2017). Validation of the French version of the health-related quality of life questionnaire for adult strabismus (AS-20). *Journal Français d’Ophtalmologie,**40*(9), 738–743. 10.1016/j.jfo.2017.04.011 29050927

[40] Ali, N., et al. (2016). Evaluation and validity of the Danish version of the adult strabismus questionnaire AS-20. *Clinical Ophthalmology,**10*, 65–69. 10.2147/OPTH.S90844 PMC4706120 26770057

[41] Wang, Z. H., et al. (2013). Development and application of the Chinese version of the adult strabismus quality of life questionnaire (AS-20): A cross-sectional study. *Health and Quality of Life Outcomes,**11*, 180. 10.1186/1477-7525-11-180 PMC4231473 24164742

[42] Marcon, G., & Pittino, R. (2017). The Italian version of the adult strabismus-20 (AS-20) questionnaire: Translation validation and reliability. *New Frontiers in Ophthalmology,**3*(5), 1–6. 10.3109/09273972.2014.932396 25005248

[43] Gothwal, V. K., et al. (2015). Measuring health-related quality of life in strabismus: A modification of the adult strabismus-20 (AS-20) questionnaire using Rasch analysis. *PLoS ONE,**10*(5), Article e0127064. 10.1371/journal.pone.0127064 PMC4444101 26011430

[44] Akbari, M. R., et al. (2015). Development of a Persian version of the adult strabismus questionnaire and evaluating the effect of strabismus surgery on health-related quality of life. *Strabismus,**23*(2), 66–72. 10.3109/09273972.2015.1025986 26158472

[45] Havstam Johansson, L., Levinsson, A., & Flodin, S. M. (2024). Translation, evaluation and validation of the adult strabismus -20 (AS-20) questionnaire in Swedish. *Clinical Ophthalmology,**18*, 3615–3625. 10.2147/OPTH.S477032 PMC11633298 39664784

[46] Mason, A., et al. (2023). Health-related quality of life in adult patients with strabismus-translation and psychometric testing of the adult strabismus questionnaire (AS-20) into Finnish. *International Journal of Environmental Research and Public Health*. 10.3390/ijerph20042830 PMC9956330 36833527

[47] Ortiz Montero, T., et al. (2023). Translation and transcultural adaptation of the AS-20 scale to measure quality of life in adults with strabismus in Colombia, a pilot study. *Archivos de la Sociedad Española de Oftalmología (English Edition),**98*(3), 142–149. 10.1016/j.oftale.2022.11.005 36577464

[48] Takahashi, S., et al. (2025). Reliability and validity of pre- and post-operative health-related quality of life in strabismus patients using the Japanese version of the adult strabismus questionnaire (AS-20). *Japanese Journal of Ophthalmology,**69*(2), 159–165. 10.1007/s10384-025-01162-x 39918703

[49] Terwee, C. B., et al. (2003). On assessing responsiveness of health-related quality of life instruments: Guidelines for instrument evaluation. *Quality of Life Research,**12*(4), 349–362. 10.1023/a:1023499322593 12797708

[50] Terluin, B., Eekhout, I., & Terwee, C. B. (2022). Improved adjusted minimal important change took reliability of transition ratings into account. *Journal of Clinical Epidemiology,**148*, 48–53. 10.1016/j.jclinepi.2022.04.018 35436522

[51] Terluin, B., et al. (2024). Estimating anchor-based minimal important change using longitudinal confirmatory factor analysis. *Quality of Life Research,**33*(4), 963–973. 10.1007/s11136-023-03577-w 38151593

[52] Terluin, B., et al. (2024). Effect of present state bias on minimal important change estimates: A simulation study. *Quality of Life Research,**33*(11), 2963–2973. 10.1007/s11136-024-03763-4 PMC11541299 39174866

[53] Zhang, Y., Xi, X., & Huang, Y. (2023). The anchor design of anchor-based method to determine the minimal clinically important difference: A systematic review. *Health and Quality of Life Outcomes,**21*(1), 74. 10.1186/s12955-023-02157-3 PMC10350268 37454099

[54] Hays, R. D., & Peipert, J. D. (2021). Between-group minimally important change versus individual treatment responders. *Quality of Life Research,**30*(10), 2765–2772. 10.1007/s11136-021-02897-z PMC8204732 34129173

